# Effect of feed conditioning time prior to pelleting on standardized ileal digestibility of amino acids and total tract digestibility of energy in diets fed to growing pigs

**DOI:** 10.1093/tas/txaf153

**Published:** 2025-11-09

**Authors:** Diego A Lopez, Hans H Stein, Matt D Miesner, Jordan T Gebhardt, Charles R Stark, Chad B Paulk

**Affiliations:** Department of Grain Science, Kansas State University, Manhattan, KS 66506, United States; Department of Animal Sciences, University of Illinois, Urbana, IL 61801, United States; Department of Diagnostic Medicine/Pathobiology, College of Veterinary Medicine, Kansas State University, Manhattan, KS 66506, United States; Department of Diagnostic Medicine/Pathobiology, College of Veterinary Medicine, Kansas State University, Manhattan, KS 66506, United States; Department of Grain Science, Kansas State University, Manhattan, KS 66506, United States; Department of Grain Science, Kansas State University, Manhattan, KS 66506, United States

**Keywords:** amino acids, conditioning, digestibility, energy, pelleting, pigs

## Abstract

Two experiments were conducted to determine the effect of increasing retention time in the conditioner prior to pelleting on digestibility of energy, protein, and amino acids (**AA**) in diets for growing pigs. Four dietary treatments were used in both experiments and consisted of a mash diet, and 3 pelleted diets that were conditioned for 30, 60, or 180 s. In Exp. 1, 12 ileal canulated barrows with an initial average body weight of 44.9 ± 2.70 kg were allotted to a triplicated 4 × 4 Latin square with 4 dietary treatments and 4 experimental periods in each square for a total of 12 replicate pigs per treatment. Each period consisted of 5 days of adaptation and 2 days of collection of ileal digesta. In Exp. 2, 12 barrows (initial average body weight: 34.1 ± 1.03 kg) were allotted to a triplicated 4 × 3 incomplete Latin square design with 4 dietary treatments and 3 experimental periods. Each period consisted of 5 d of adaptation and 5 d of collection of feces and urine. Pigs were individually housed in metabolism crates and feces, and urine were collected. Results of Exp. 1 indicated an overall treatment effect (*P *< 0.05) on standardized ileal digestibility (**SID**) of crude protein and all AA except Lys, Met, and Trp. The SID of crude protein, Arg, Leu, Thr, Ala, Ser, and Tyr was greater (*P *< 0.05) in all pelleted diets compared with the mash diet. The SID of His, Ile, Phe, Val, Asp, and Glu was less (*P *< 0.05) in the mash diet compared with the pelleted diets conditioned for 60 s or 180 s. The SID of Cys was less (*P *< 0.05) in the mash diet compared with the pelleted diet conditioned for 180 s. Increasing the retention time in the conditioner from 30 to 180 s increased (linear; *P *< 0.05) the SID of CP and most AA. Results of Exp. 2 demonstrated that pelleted diets had greater (*P *< 0.05) apparent total tract digestibility (**ATTD**) of gross energy compared with the mash diet, but no treatment effects were observed for ATTD of dry matter or on digestible energy, metabolizable energy or the metabolizability of digestible energy. These results indicate that increasing conditioning time up to 180 s improved AA digestibility for most AA and pelleting also increased ATTD of gross energy.

## Teaser

Pelleting improved energy, crude protein, and amino acids digestibility, and increasing conditioning time improved amino acid digestibility further.

## Introduction

Conditioning feed with steam prior to pelleting is a key process to prepare the feed for pellet formation in the pellet die (Wang et al. 2019). Proper mash feed conditioning is the first major step of the pelleting process and is necessary to achieve optimum production rates, pellet quality, energy usage, and die and roll life. Increasing the amount of steam used in the conditioning process will in turn result in increased conditioning temperature and moisture added to the feed. Addition of heat and moisture has been attributed to changing the physicochemical properties of the feed, typically resulting in improved binding properties between particles (Thomas and van der Poel 1996), which may increase nutrient digestibility (Svihus and Zimonja 2011).

Parameters that can be adjusted during feed conditioning prior to pelleting include steam addition (temperature and moisture), retention time, and steam pressure. Conditioner retention time is influenced by the length of the conditioner, the angle and placement of the mixing paddles, and the operating speed. When steam is added to the conditioner, moisture absorption occurs at a slower rate than heat transfer (Thomas and van der Poel 2020), which supports the hypothesis that longer retention times may influence pellet durability and nutrient digestibility by promoting physicochemical changes through increased moisture absorption.

Increasing the retention time during conditioning has also been considered a strategy to reduce microbial contamination of feed (Boroojeni et al. 2016; Cochrane et al. 2017; Boltz et al. 2019). Although research has shown the capacity of feed to transmit pathogens such as viruses and bacteria, temperature and retention time sufficient to neutralize those pathogens are or can be achieved during conditioning (Cochrane et al. 2017). Increasing the temperature during conditioning can be an effective method of control, however, it might lead to heat damage of nutrients (González-Vega et al. 2011). As an alternative, using lower temperatures in combination with longer retention times may neutralize pathogens while minimizing nutrient damage (Boroojeni et al. 2016; dos Santos et al. 2020). To increase feed retention time in the conditioner, operators can adjust the paddle configuration to reduce forward movement, reduce conditioner shaft speed, or reduce feed production rate to decrease the flow rate of feed through the conditioner. Hygienizers or double- and triple-pass conditioners can also be used to increase retention time during conditioning. These systems provide greater flexibility to control the duration of feed exposure to high temperature and moisture.

Although it has been suggested that the ideal retention time is in the range of 30 to 90 s, it is estimated that most conditioners range between 5 and 10 s (Schofield and American Feed Industry Association 2005). Therefore, feed mills may explore opportunities to maximize feed retention time in the conditioner to improve pellet durability and microbial control. Additionally, an increase in digestibility of energy and nutrients was observed when retention time increased from 3 to 20 s in pelleted diets fed to broilers (dos Santos et al. 2020). However, to our knowledge, no research has been conducted to determine the effect of extended retention time on nutrient and energy digestibility. Therefore, the objective of this work was to test the hypothesis that extended retention time of feed in the conditioner prior to pelleting will increase the standardized ileal digestibility (**SID**) of amino acids (**AA**) and the apparent total tract digestibility (**ATTD**) of energy of diets fed to growing pigs.

## Materials and methods

The Institutional Animal Care and Use Committee at Kansas State University approved the protocol for two experiments. Castrated male pigs (241 × 600, DNA Genetics, Columbus, NE, United States) were used in both experiments. Pigs were housed individually in metabolism crates (1.50 m × 0.56 m) that were equipped with solid panels on the sides, a feeder, a water cup drinker, and metal diamond-slatted flooring.

### Diet and diet preparation

A diet based on corn, soybean meal, and distillers dried grains with solubles was formulated (Tables 1 and 2) to meet or exceed all nutrient requirements for pigs between 40 and 65 kg (NRC 2012). The diet was manufactured at the O.H. Kruse Feed Technology and Innovation Center in Manhattan, KS, United States, following their standard procedures. A total of 2449 kg of a basal diet was mixed and used for both experiments. Four separate batches were mixed using a twin-shaft counterpoise mixer (Hayes and Stolz, model TRDB63-0152, Fort Worth, TX, United States). The mixing time was 60 s for dry ingredients and an extra 180 s after adding liquid ingredients. After mixing, one batch of 455 kg of feed was kept as mash and the remaining 3 batches of 664 kg of mixed feed were used in the manufacturing of pelleted treatments.

**Table 1.** Ingredient composition of experimental diets (as-fed basis).^a^

Item	Basal diet
**Ingredient, %**	
** Corn**	59.62
** Soybean meal**	16.14
** Distillers dried grains with solubles**	20.00
** Soybean oil**	1.00
** Calcium carbonate**	0.95
** Monocalcium phosphate, 21% P**	0.30
** Sodium chloride**	0.40
** L-Lysine-HCl**	0.45
** DL-Methionine**	0.05
** L-Threonine**	0.12
** L-Tryptophan**	0.05
** L-Valine**	0.02
** Trace mineral premix** ^b^	0.15
** Vitamin premix** ^c^	0.25
** Phytase concentrate** ^d^	0.01
** Titanium dioxide**	0.50
** Total**	100.00
**Calculated analysis**	
** Standardized ileal digestible AA, %**	
** Lysine**	1.00
** Isoleucine: lysine**	61
** Leucine: lysine**	156
** Methionine: lysine**	33
** Methionine and cysteine: lysine**	58
** Threonine: lysine**	70
** Tryptophan: lysine**	20.3
** Valine: lysine**	72
** Histidine: lysine**	42
** Total lysine, %**	1.16
** Metabolizable energy, kcal/kg**	3,803
** Crude protein, %**	18.70
** Ca, %**	0.57
** P, %**	0.45

a A basal diet was formulated and divided into 4 batches. One batch was used without further processing, but three batches were pelleted following conditioning at 30, 60, or 180 s).

b Provided per kg of diet: 110 mg of Zn from zinc sulfate; 110 mg of Fe from iron sulfate; 33 mg of Mn from manganese oxide; 17 mg of Cu from copper sulfate; 0.30 mg of Se from sodium selenite; 0.30 mg of I from ­calcium iodate.

c Provided per kg of diet: 4,134 IU vitamin A; 1,653 IU vitamin D_3_; 44 IU vitamin E; 3 mg vitamin K; 0.03 mg vitamin B_12_; 50 mg of niacin; 28 mg pantothenic acid; 8 mg riboflavin.

d Quantum Blue 10 g (AB Vista, Marlborough, Wiltshire, UK) contained 10,000 FTU/g. At 0.01% inclusion, complete diets were expected to contain 500 FTU/kg with an estimated release of 0.10% STTD P.

**Table 2.** Analyzed composition of experimental diets (as-fed basis).

		Pelleted diets—retention time, s		
Item,	Mash diet	30	60	180
**Gross energy, kcal/kg**	4,101	3,963	3,986	3,998
**Dry matter, %**	87.89	87.47	87.44	87.51
**Crude protein, %**	19.29	19.04	19.14	19.07
**Crude fat, %**	3.04	4.15	4.18	4.03
**Crude fiber, %**	3.06	4.06	4.33	5.09
**Ash, %**	5.81	4.98	5.06	5.04
**Indispensable AA, %**				
** Arginine**	1.07	1.02	0.99	1.02
** Histidine**	0.51	0.49	0.48	0.49
** Isoleucine**	0.82	0.77	0.76	0.80
** Leucine**	1.87	1.80	1.78	1.87
** Lysine**	1.32	1.25	1.18	1.22
** Methionine**	0.43	0.34	0.34	0.35
** Phenylalanine**	0.95	0.90	0.89	0.94
** Threonine**	0.80	0.80	0.76	0.80
** Tryptophan**	0.22	0.22	0.21	0.22
** Valine**	0.98	0.93	0.90	0.94
**Dispensable AA, %**				
** Alanine**	1.10	1.07	1.06	1.10
** Aspartic acid**	1.61	1.56	1.53	1.60
** Cysteine**	0.35	0.33	0.32	0.34
** Glutamic acid**	3.29	3.14	3.12	3.28
** Glycine**	0.77	0.74	0.72	0.74
** Serine**	0.79	0.75	0.74	0.78
** Tyrosine**	0.68	0.64	0.63	0.66

The mash diet was mixed and sacked off after mixing without further processing. To prepare the pelleted diets, the mash feed was conditioned for approximately 30, 60, or 180 s at a target temperature of 85 °C in a single-pass conditioner. To accomplish the desired retention time, the paddles of the conditioner were set at an angle of 10°, the feeder flow to the conditioner was adjusted to 10%, and the speed of the conditioner was adjusted to 85%, 60%, or 10%. Following condition, each of the 3 diets was pelleted on a 100 HP pellet mill (CPM, model 3016-4 Master, Crawfordsville, IN, USA) equipped with a 4.8 × 45.0 mm die. Each diet was pelleted in a single run that took approximately 80 min. During pelleting, the temperature in the conditioner was monitored and recorded every 20 minutes (Table 3).

**Table 3.** Feed processing and pellet quality of pelleted diets.^a^^,^^b^

	Pelleted diets—retention time, s		
Item	30	60	180
**Production rate, kg/h**	504	487	472
**Conditioning temperature, °C**	83.8	84.0	83.1
**Pellet durability index (PDI), %^c^**	81.6	84.8	88.7

a Treatments were pelleted using a 5-ton 100-horsepower pellet mill (Model PM3016-4, CPM, Crawfordsville, IN, United States) equipped with a 4.8 × 45-mm die.

b The values for production rate and conditioning temperatures are the averages of four measurements taken at evenly spaced intervals over the duration of the pellet run.

c Holmen NHP100 (Norfolk, United Kingdom) for 60 s; Samples were analyzed in triplicate.

Once pelleted, diets were analyzed for pellet quality using the Holmen forced-air method (TekPro Ltd, model NHP 100, Norfolk, United Kingdom; Evans 2023). Prior to analysis, samples were stored in commercial paper feed sacks. Each sample was analyzed in duplicate, and results were averaged. Collected pellet samples were sifted with a U.S. No. 5 (3.9 mm) sieve to allow for the separation of fines and pellets. The pellet durability was then assessed using the NHP 100 (TekPro Ltd, Norfolk, United Kingdom) pellet tester. The following equation was used to calculate the pellet durability index (**PDI**; S269.5; ASAE 2012):


$$\mathrm{PDI}\;(\%)=\frac{\mathrm{Weight}\;\mathrm{of}\;\mathrm{recovered}\;\mathrm{pellets}}{\mathrm{Intitial}\;\mathrm{weight}\;\mathrm{of}\;\mathrm{pellets}}\times 100$$

### Experiment 1: amino acid digestibility

A total of 12 barrows (average initial body weight: 44.95 ± 2.70 kg) were surgically equipped with a T-cannula in the distal ileum (Stein et al. 1998). The animals were allotted to a triplicated 4 × 4 Latin square design (Kim and Stein 2009) with four periods of 7 d and 4 dietary treatments. Therefore, each diet was fed to 3 pigs in each period for a total of 12 replicate pigs per dietary treatment for the 4 periods. Pigs were fed 3.0 times the maintenance requirement for metabolizable energy (**ME**; ie 197 kcal/kg body weight^0.60^; NRC 2012) and had free access to water throughout the experiment. The daily feed allowance was divided and fed in two equal meals at 0800 and 1400 h.

The first five days of each period were considered the adaptation period to the diet (Adeola 2001), with the ileal sample collection taking place on days 6 and 7. On each collection day, samples were collected for 8 h by removing the lid of the cannula, attaching a plastic bag to the barrel of the cannula, and securing it with a plastic cable tie. The digesta flowing into the bag were collected every 30 min or every time the bag was full. Digesta samples were immediately stored at −20 °C to prevent bacterial degradation of AA.

Diets were bagged, and a sample was collected by probing each bag and combining into one composite sample. Digesta samples were thawed, subsampled, lyophilized, and ground in preparation for laboratory analysis. Samples of diets and ileal digesta were analyzed for dry matter (**DM**; method 930.15; AOAC 2019) and nitrogen using the combustion method in a LECO analyzer (method 990.03; AOAC 2019). Crude protein (**CP**) was calculated as nitrogen × 6.25. Diet and ileal digesta samples were also analyzed for AA (method 982.30 E [a, b, c]; AOAC 2019) and titanium was quantified following the method described by Myers et al. (2004). Diet samples were also analyzed for ash (method 942.05; AOAC 2019), crude fiber (method 978.10; AOAC 2019), and crude fat (method 920.39; AOAC 2019).

Apparent ileal digestibility (**AID**) and SID of CP and AA were calculated for the four dietary treatments as described by Stein et al. (2007). The values for the basal endogenous losses of CP and AA used to calculate SID were obtained from Adeola (2016).

### Experiment 2: energy

Twelve barrows (average initial body weight: 34.11 ± 1.03 kg) were allotted to a 4 × 3 incomplete Latin square design with 4 treatments and three periods of 13 d, for a total of nine replicate pigs per dietary treatment. During each experimental period, the initial first five days were considered an adaptation period to the diets and the adaptation period was followed by 5 d of collection of feces and urine. Feeding was managed as described in Exp. 1.

During collection, a urine pan and a screen were installed underneath the floor of the crates to allow for the total, but separate, collection of fecal and urine samples. Fecal samples were collected following the marker-to-marker approach (Adeola 2001). In short, an indigestible marker was included in the meal of the morning of the first day of collection, and the collection of feces started the moment the marker was spotted in the feces. A second indigestible marker was included in the meal on the morning of the last day of collection, and then the fecal collection stopped the moment the second marker was spotted in the feces. Unconsumed feed left in the feeder during the collection period was weighed, collected, and recorded. Orts were collected as well. Urine was collected every morning during the collection period. A bucket with 50 mL of 6 *N* HCl to prevent volatilization was placed underneath the urine pan under each crate and covered with cheesecloth. The buckets with the urine content were weighed daily, and 10% of the content was subsampled. Both feces and urine samples were immediately stored at −20 °C. After the subsample of urine was taken, the remaining urine was discarded and HCl was added to the buckets again.

At the conclusion of the experiment, urine samples were thawed and mixed within animal and diet. A subsample was then taken for chemical analysis. Fecal samples were dried at 55 °C in a forced-air oven (Jacobs et al. 2011), finely ground, mixed, and subsampled. Urine samples were filtered and lyophilized. Feed, urine, and fecal samples were analyzed for gross energy using bomb calorimetry (Parr 6200, Parr Instruments Company, Moline, IL, USA). Feed and fecal samples were analyzed for DM (Method 930,15; AOAC 2019). Following analysis of diets, fecal samples, and urine samples, ATTD of gross energy and DM was calculated and digestible energy (**DE**) and ME were calculated for each diet (Adeola 2001).

### Statistical analyses

Data were analyzed using the GLIMMIX procedure of SAS (SAS Institute Inc., Cary, NC, United States) using normal distribution and considering pig as the experimental unit. Diet was the fixed effect and pig and period were random effects. Least squares means were calculated for each independent variable and means were separated using the PDIFF option. Studentized residuals were calculated for each observation. Observations with a studentized residual outside of ± 3 were considered outliers and removed. Contrast statements were used to test linear effects of increasing retention time in the conditioner in pelleted treatments; therefore, the mash diet was not included in the contrasts. Treatment differences were considered significant at *P *≤ 0.05 and marginally significant at 0.05 < *P *≤ 0.10.

## Results

Because the four treatment diets were produced using the same ingredient composition, only small variations in chemical composition were observed among diets (Table 2). However, differences were observed in the concentration of GE in the mash diet compared with the pelleted diets. Additionally, differences in the concentration of Lys and Met were observed between the mash diet compared with the pelleted diets, however, these differences were considered to be within the range of analytical or sampling variability.

The expected conditioning temperature was 85 °C. Actual conditioning temperatures averaged 83.6 °C. Pellet mill production rates averaged 488 kg/hr (Table 3). Increasing the retention time of feed in the conditioner from 30 to 180 s numerically improved PDI from 81.6% to 88.7%.

No overall treatment effects were observed for the AID of Lys, Met, or Trp (Table 4). The AID of DM, CP, Thr, Leu, and Ala in pelleted diets, regardless of retention time, was greater (*P *< 0.05) than in the mash diet. The AID of Arg, His, Ile, Phe, Val, Asp, Glu, Ser, Tyr, mean indispensable AA, mean dispensable AA, and mean total AA was greater (*P *< 0.05) in pelleted diets with retention times of 60 and 180 s compared with the mash diet; however, no differences were observed between the 30 s and the mash diets for the AID of these AA. The AID of Cys and Gly was greater (*P *< 0.05) only in the diet pelleted after condition for 180 s compared with the mash diet. A linear (*P *< 0.05) increase was observed in the AID of DM and most AA except for Lys and Trp as retention time in the conditioner increased from 30 to 180 s.

**Table 4.** Apparent ileal digestibility (AID) of dry matter, crude protein and amino acid (AA) in experimental diets.^1^

		Retention time, s				*P* <,	
Item, %	Mash	30	60	180	SEM	Treatment	Linear^2^
** Dry matter**	63.5^b^	65.9^a^	66.3^a^	68.0^a^	0.71	0.001	0.014
** Crude protein**	64.7^b^	71.3^a^	73.4^a^	73.9^a^	1.01	0.001	0.122
**Indispensable AA**							
** Arginine**	82.4^c^	84.2^bc^	85.3^ab^	86.1^a^	0.71	0.001	0.013
** Histidine**	75.1^c^	76.3^bc^	78.1^ab^	79.1^a^	0.89	0.001	0.005
** Isoleucine**	73.2^c^	75.5^bc^	77.5^ab^	78.9^a^	1.10	0.001	0.004
** Leucine**	76.2^c^	79.6^b^	81.5^ab^	82.7^a^	0.99	0.001	0.004
** Lysine**	78.6	79.5	78.7	79.8	0.99	0.488	0.451
** Methionine**	83.9	83.4	84.8	85.4	0.88	0.052	0.026
** Phenylalanine**	74.8^c^	77.3^bc^	79.3^ab^	80.7^a^	1.02	0.001	0.004
** Threonine**	67.0^b^	70.2^a^	71.0^a^	72.9^a^	1.05	0.001	0.017
** Tryptophan**	79.6	80.0	80.8	81.5	1.00	0.252	0.150
** Valine**	70.4^c^	73.0^bc^	74.4^ab^	76.0^a^	1.17	0.001	0.013
**Mean**	75.8^c^	77.9^bc^	79.1^ab^	80.4^a^	0.94	0.001	0.012
**Dispensable AA**							
** Alanine**	70.3^c^	74.2^b^	76.4^ab^	77.6^a^	1.20	0.001	0.009
** Aspartic acid**	68.6^c^	70.8^bc^	72.8^ab^	74.2^a^	1.04	0.001	0.008
** Cysteine**	58.8^b^	57.7^b^	61.0^ab^	64.8^a^	1.46	0.002	0.001
** Glutamic acid**	75.2^c^	76.7^bc^	78.7^ab^	80.5^a^	1.05	0.001	0.005
** Glycine**	52.3^b^	53.6^ab^	55.2^ab^	58.4^a^	1.67	0.022	0.035
** Serine**	72.2^c^	74.4^bc^	76.0^ab^	77.5^a^	0.92	0.001	0.002
** Tyrosine**	76.0^c^	78.2^bc^	79.7^ab^	81.1^a^	0.90	0.001	0.002
**Mean**	70.4^c^	72.4^bc^	74.3^ab^	76.3^a^	1.06	0.001	0.003
**Total AA**	73.0^c^	75.0^bc^	76.6^ab^	78.2^a^	0.98	0.001	0.005

Means within a row lacking a common superscript letter (a to c) are different (*P *< 0.05).

1 *N* = 12.

2 Linear effect of increasing conditioning time during pelleting (ie 30, 60, and 180 s).

No overall treatment effects were observed for the SID of Lys or Trp (Table 5). All pelleted diets had a greater (*P *< 0.05) SID of CP, Thr, Arg, Leu, Ala, Ser, and Tyr compared with the mash diet. Diets pelleted with conditioner retention times of 60 or 180 s had greater (*P *< 0.05) SID of Arg, His, Ile, Leu, Phe, Val, Ala, Asp, Glu, Ser, Tyr, mean dispensable AA, mean indispensable AA, and mean total AA compared with the mash diet. Increasing the retention time in the conditioner from 30 to 180 s increased (linear; *P *< 0.05) the SID of CP and most AA, except for Lys and Trp.

**Table 5.** Standardized ileal digestibility of crude protein and amino acids (AA) in experimental diets.^1,2^

		Retention time, s				*P <*,	
Item, %	Mash	30	60	180	SEM	Treatment	Linear^3^
** Crude protein**	73.5^b^	79.2^a^	81.2^a^	81.9^a^	1.01	0.001	0.044
**Indispensable AA**							
** Arginine**	87.3^c^	89.2^b^	90.5^ab^	91.1^a^	0.71	0.001	0.016
** Histidine**	78.0^c^	79.3^bc^	81.1^ab^	82.1^a^	0.89	0.001	0.006
** Isoleucine**	76.4^c^	78.9^bc^	80.9^ab^	82.2^a^	1.10	0.001	0.006
** Leucine**	78.5^c^	82.0^b^	83.9^ab^	85.1^a^	0.99	0.001	0.005
** Lysine**	81.2	82.3	81.7	82.7	0.99	0.436	0.441
** Methionine**	86.2^a^	86.2^a^	87.6^a^	88.1^a^	0.89	0.027	0.034
** Phenylalanine**	77.8^c^	80.4^bc^	82.4^ab^	83.6^a^	1.02	0.001	0.005
** Threonine**	72.7^b^	75.9^a^	76.9^a^	78.6^a^	1.05	0.001	0.022
** Tryptophan**	84.8	85.1	86.2	86.7	1.00	0.225	0.176
** Valine**	74.6^b^	77.3^ab^	78.9^a^	80.3^a^	1.17	0.001	0.017
**Mean**	79.2^c^	81.5^bc^	82.8^ab^	83.9^a^	0.94	0.001	0.016
**Dispensable AA**							
** Alanine**	74.8^c^	78.9^b^	81.1^ab^	82.2^a^	1.20	0.001	0.012
** Aspartic acid**	72.7^c^	75.0^bc^	77.1^ab^	78.3^a^	1.04	0.001	0.011
** Cysteine**	63.1^b^	63.2^b^	65.7^ab^	69.2^a^	1.46	0.002	0.001
** Glutamic acid**	77.7^c^	79.4^bc^	81.3^ab^	83.0^a^	1.05	0.001	0.007
** Glycine**	69.0^b^	70.9^ab^	72.9^ab^	75.6^a^	1.67	0.011	0.021
** Serine**	79.4^c^	81.9^b^	83.7^ab^	84.8^a^	0.92	0.001	0.006
** Tyrosine**	80.6^c^	83.0^b^	84.5^ab^	85.7^a^	0.90	0.001	0.004
**Mean**	79.2^c^	81.5^bc^	83.5^ab^	85.1^a^	1.06	0.001	0.006
**Total AA**	79.2^c^	81.5^bc^	83.2^ab^	84.6^a^	0.98	0.001	0.008

Means within a row lacking a common superscript letter (a to c) are different (*P *< 0.05).

1 *N* = 12.

2 Values for standardized ileal digestibility were obtained with the correction of apparent ileal digestibility values for basal endogenous losses of each individual AA and crude protein. These values were obtained from Adeola (2016) as (g/kg of dry matter intake): Crude protein, 17.28; Arg, 0.59; His, 0.17; Ile 0.30; Leu, 0.50; Lys, 0.40; Met, 0.11; Phe, 0.32; Thr, 0.52; Trp, 0.13; Val, 0.46; Ala, 0.57; Asp 0.75; Cys, 0.17; Glu, 0.94; Gly, 1.46; Ser, 0.65; Tyr, 0.35.

3 Linear effect of increasing conditioning time during pelleting (ie 30, 60, and 180 s).

No differences were observed in the ATTD of DM, concentrations of DE and ME, or the metabolizability of DE (ie ME: DE; Table 6). However, the ATTD of GE was greater (*P *< 0.05) in the pelleted diets compared with the mash diet.

**Table 6.** Digestible energy (DE), metabolizable energy (ME), and apparent total tract digestibility (ATTD) of dry matter and gross energy.^1^

		Retention time, s				*P* <,	
Item,	Mash	30	60	180	SEM	Treatment	Linear^2^
**ATTD of dry matter, %**	86.76	87.79	87.42	87.50	0.32	0.135	0.676
**ATTD of gross energy, %**	85.14^b^	87.48^a^	87.06^a^	87.66^a^	0.63	0.001	0.545
**DE, kcal/kg of dry matter**	3973	3963	3969	4004	28.66	0.437	0.112
**ME, kcal/kg of dry matter**	3781	3787	3767	3812	43.26	0.671	0.337
**ME: DE, %**	95.07	95.66	94.92	95.19	0.60	0.655	0.683

Means within a row lacking a common superscript letter (a and b) are different (*P *< 0.05).

1 *N* = 9.

2 Linear effect of increasing conditioning time during pelleting (ie 30, 60, and 180 s).

## Discussion

Although it has been suggested that the ideal retention time in the conditioner is in the range of 30 to 90 s, most conditioners have an estimated retention time shorter than 30 s (Schofield and American Feed Industry Association 2005; Behnke 2014; Dunmire et al. 2021). However, retention time in the conditioner can be increased. The angle of paddles is usually factory set at 30 to 45° forward angle, which can be modified to a lesser angle to reduce the speed at which feed will move through the conditioner. The closer the paddles are to be perpendicular to the shaft, the greater retention time can be accomplished (Schofield and American Feed Industry Association 2005; Behnke 2014). Modifying the shaft speed can also alter retention time. However, shaft speed works in combination with the paddle angle and there are different combinations that might lead to different retention times, and achieving the desired retention time as a result of the combined effects of shaft speed and paddle angle is a trial-and-error process (Schofield and American Feed Industry Association 2005; Behnke 2014). With the equipment available to prepare diets for the present experiments, the desired retention times were accomplished by reducing the shaft speed of the conditioner, adjusting the angle of the paddles in the conditioner, and reducing the amount of feed flowing into the conditioner. The first retention time accomplished was 180 s, due to the degree of difficulty to accomplish this time. Once the paddles and the flow of feed into the conditioner were adjusted to achieve 180 s of retention, these settings remained constant across all pelleting runs. To achieve the remaining targets of retention time, the only parameter that was modified was the conditioner shaft speed, therefore, it was assumed that all other conditioning parameters (ie temperature, steam quality, pressure, etc.) were similar across treatments.

During pelleting, the exposure of feed to heat and moisture creates conditions for changes that potentially affect its nutritional value as described below. Improvements in ileal digestibility of CP and AA when pelleting swine diets have been reported (Rojas et al. 2016; Dunmire et al. 2024; Lee et al. 2025). In the case of protein, there is a degree of denaturation of protein and inactivation of antinutritional factors that can lead to these potential improvements in AA digestibility. However, if ingredients or complete feed are over processed, Maillard reactions may occur which could result in negative effects on protein digestibility (González-Vega et al. 2011).

The chemical reactions involved in Maillard reactions can render AA indigestible due to the link of its amino group with a reducing sugar (Martins et al. 2000). Because Maillard reactions require a free amino group to occur, amino acids differ in their susceptibility. For example, lysine is particularly reactive due to the presence of both an α-amino group, like all amino acids, and an additional ε-amino group (Martins et al. 2000). Tryptophan is also susceptible due to the nitrogen within its indole ring. As a result, the Maillard reaction might reduce digestibility of Lys and Trp before affecting other amino acids, due to their greater susceptibility to heat-induced reactions compared with other AA (Ajandouz and Puigserver 1999; Salazar-Villanea et al. 2018). The observation that there were differences between mash and pelleted diets for the digestibility of Lys and Trp contrast with previous experiments (Rojas et al. 2016; Dunmire et al. 2024; Lee et al. 2025) where AID or SID of Lys and Trp increased with pelleting. However, because no improvements or reduction in the digestibility of Lys or Trp were observed, it is hypothesized that the positive effects of pelleting on Lys or Trp digestibility were offset by the negative effects of the Maillard reactions. Under the conditions of the present experiment, the slower production rate during pelleting might have resulted in prolonged residence time of the feed in the pellet die, where higher temperatures are reached due to friction and pressure. The extended exposure to heat may also have promoted Maillard reactions to a greater extent compared with previous studies, and therefore, potentially explaining the differences observed in the AID and SID of Lys and Trp compared with the mash diet.

Protein denaturation, particularly changes in tertiary structure, can occur during processes involving high temperatures and the presence of moisture in the environment. The temperature at which denaturation occurs is directly influenced by environmental moisture; proteins exposed to high moisture can denaturate at temperatures as low as 60 to 70 °C, whereas proteins exposed to lower moisture conditions require higher temperatures that can reach 100 to 200 °C (Adams 1991; Svihus and Zimonja 2011; Zhang et al. 2022). During pelleting, the use of steam, increases the feed moisture concentration 3% to 6% (Schofield and American Feed Industry Association 2005), while also increasing the temperature during conditioning. This increase in the temperature and the presence of moisture indicate the capacity of pelleting of promoting protein denaturation, which can partially explain the positive effect of pelleting on SID of CP and AA.

Although increasing the conditioning temperature by adding steam increases the amount of moisture added to the feed, increasing the retention time allows more time for that moisture and heat to be absorbed and penetrate feed particles. Therefore, increasing the retention time during conditioning resulted in increased AID and SID of most AA. The observation of increased AID and SID of AA in the diets pelleted using 180 s of retention time, compared with the 30 s retention time, may be a result of the additional time that the moisture in the conditioner could penetrate the feed and enhance the reactions produced by the heat treatment. Water diffusion is 100 times slower than heat diffusion (Thomas and van der Poel 2020), resulting in a longer time needed for a particle to be hydrated even in the presence of sufficient moisture, compared with the time needed to reach a higher temperature. Moisture is not only one of the most important contributors to the binding of particles in the pellet (Thomas and van der Poel 1996) which results in a more durable pellet (Cutlip et al. 2008; Abdollahi et al. 2010; 2013; Evans et al. 2021), but water is also an important factor in the reactions that might result in increased CP and AA digestibility. It is hypothesized that the extended residence of the feed in the conditioner contributed to a greater absorption of moisture in the feed, which in turn it might have facilitated protein denaturation, inactivation of antinutritional factors, and starch gelatinization during the formation of the pellet that resulted in the observed increased digestibility of CP and AA.

The increase in the ATTD of GE in the pelleted diets compared with the meal diet is in agreement with published data (Wondra et al. 1995; Le Gall et al. 2009; Rojas et al. 2016; Lee et al. 2024). Gelatinization of starch is considered the main reason for the increase in ATTD of GE in diets fed to pigs (Lancheros et al. 2020). The high heat and moisture conditions during pelleting can promote the process of gelatinization in the grains in the feed. Due to the swelling and leaking of starch out of the granules (Hoseney 1986), starch becomes more available for the endogenous enzymes needed for digestion, which may result in increased absorption of glucose in the small intestine. Changes in conditioning temperature and time may contribute to increased starch gelatinization in pelleted diets (Lewis et al. 2015), but conditioning temperature may be less important than the effect of the feed being pushed through the pellet die where friction temperature is generated. Under the conditions of the present experiment, increasing the retention time in the conditioner did not translate into an increase in the ATTD of GE, which could be an indication of minimal change in the concentration of gelatinized starch with increasing conditioning retention time. Limited research has been conducted to determine the effect of pelleting on the ATTD of fat (Le Gall et al. 2009; Lee et al. 2024) although the contribution of fat to the total GE energy of diets is significant due to its caloric density. An increase in ATTD of fat in pelleted diets compared with mash diets has been observed (Lee et al. 2024). However, due to the low inclusion of fat in the diets used in this experiment it is unlikely this was a major contributor to the increased ATTD of GE.

The results observed for DE and ME contrast with reported values, indicating increased DE and ME in pelleted diets compared with mash diets (Rojas et al. 2016; Lee et al. 2024). However, results in the present experiment may have been influenced by the initial concentration of GE in the treatment diets. The mash diet had a greater concentration of GE compared with the pelleted diets, which in turn results in greater concentrations of DE and ME. This observation can be partially explained by mixing, sampling, and analysis variation. Additionally, a possible explanation for the lower GE in pelleted diets could be explained by moisture gain during conditioning, however, the concentrations of DM did not differ between treatments. Although the differences in ATTD of GE between the mash treatment and the pelleted treatments were significant, the difference is not greater than 2.5% units of ATTD, whereas the difference in GE concentrations is greater than 100 kcal/kg. This results in similar concentrations of DE and ME regardless of the greater ATTD of GE observed in pelleted diets. Furthermore, the observation of no differences in the metabolizability of DE among treatments can be an explanation for the observation of no differences on the concentrations of DE and ME across pelleted treatments. This indicates that although the ATTD of GE was greater in pelleted diets compared with mash diets, it did not influence the capacity of the pigs to further utilize the energy once it was absorbed.

In conclusion, extended retention times in the conditioner prior to pelleting increased the AID and SID of CP and most AA. In contrast, the ATTD of GE was not affected by the extended conditioning time, but it was improved by pelleting. These results indicate that increasing retention time as a strategy to enhance pellet quality or reduce microbial load can be implemented without compromising the digestibility of energy or nutrients.

## List of abbreviations:

AA: amino acid
AID: apparent ileal digestibility
ATTD: apparent total tract digestibility
CP: crude protein
DE: digestible energy
DM: dry matter
ME: metabolizable energy
PDI: pellet durability index
SID: standardized ieal digestibility
